# Effects of Sodium Hexametaphosphate on the Gel Properties and Structure of Glutaminase-Transaminase-Crosslinked Gelatin Gels

**DOI:** 10.3390/foods14132175

**Published:** 2025-06-21

**Authors:** Junliang Chen, Xia Ding, Weiwei Cao, Xinyu Wei, Xin Jin, Qing Chang, Yiming Li, Linlin Li, Wenchao Liu, Tongxiang Yang, Xu Duan, Guangyue Ren

**Affiliations:** 1 College of Food and Bioengineering, Henan University of Science and Technology, Luoyang 471023, China; chenjl2020@haust.edu.cn (J.C.); dx18137212997@163.com (X.D.); wxy15824994023@163.com (X.W.); jinxin20000212@163.com (X.J.); 13017609579@163.com (Q.C.); 13323639190@163.com (Y.L.); linlinli2020@126.com (L.L.); wen_chaoliu@163.com (W.L.); txyamy@163.com (T.Y.); duanxu_dx@163.com (X.D.); guangyueyao@163.com (G.R.); 2 Henan Province Engineering Research Center of Agricultural Products Processing Equipment, Luoyang 471000, China; 3 Henan Province Engineering Technology Research Center of Agricultural Product Drying Equipment, Luoyang 471000, China

**Keywords:** sodium hexametaphosphate, glutaminase transaminase, gelatin gel, rheological properties

## Abstract

Gelatin is a commonly used protein-based hydrogel. However, the thermo-reversible nature of gelatin makes it unstable at physiological and higher temperatures. Therefore, this study adopted phosphates and glutaminase transaminase (TG) to modify gelation and studied the effects of combining sodium hexametaphosphate (SHP) and TG on the structure and gel properties of TG-crosslinked gelatin. This study focused on the effects of different SHP concentrations (0, 0.4, 0.8, 1.2, 1.6, 2.0, 2.4, 2.8 mmol/L) on the water distribution, textural properties, rheological properties, and microstructure of the TG-crosslinked gelatin gels. Results showed that the free water content in the TG-crosslinked gelatin gel declined with the increasing SHP addition when the concentration of SHP was kept below 2.0 mmol/L. The gel of TG-crosslinked gelatin at the SHP concentration of 1.6 mmol/L exhibited the highest hardness (304.258 g), chewiness (366.916 g) and η_50_. All the TG-crosslinked gelatin gels with SHP modification were non-Newtonian pseudoplastic fluids. The G′ and G″ of TG-crosslinked gelatin increased before the SHP concentration reached 1.6 mmol/L, and the TG-crosslinked gelatin with 1.6 mmol/L SHP exhibited the largest G″ and G′. The fluorescence intensity of TG-crosslinked gelatin with SHP concentration above 1.6 mmol/L decreased with the increasing SHP concentration. SHP modified the secondary structure of TG-crosslinked gelatin gels. The gel of TG-crosslinked gelatin with the SHP concentration of 1.6 mmol/L exhibited a porous, smooth, and dense network structure. This research provides references for modifying gelatin and the application of gels in the encapsulation of bioactive ingredients and probiotics.

## 1. Introduction

Hydrogels have high content of water as 3D network materials, which are formed by the crosslinking of polymer chains with abundant hydrophilic groups through physical and chemical methods [1]. Therefore, hydrogels have been widely applied in various areas, such as self-healing materials and drug delivery [2,3]. The primary components of hydrogels include natural polymers and synthetic polymers based on their source. Natural hydrogels offer higher safety, better biocompatibility, and biodegradability compared to synthetic hydrogels [1]. Protein-based gels are a type of natural hydrogel, and gelatin is a commonly used protein-based hydrogel with the advantages of transparency and insolubility in cold water [4]. However, the thermo-reversible nature of gelatin makes it unstable at physiological and higher temperatures, limiting its application in food processing [5]. Therefore, it is necessary to adopt a modification method to change the gelatin structure to broaden its application range in the food industry [6]. The modified gelatin can be used as a thickening agent, emulsifier, and foaming agent to improve the quality of dairy products, candies, and meat products. Additionally, the modified gelatin can also be made into edible films to provide excellent gas and moisture barrier properties [6].

Phosphorylation could effectively improve protein function [7]. Phosphorylation could replace the hydroxyl groups on gelatin with phosphate groups to form phosphate ester bonds, which further enhances the hydrophilicity of gelatin and affects the solubility and gelation behavior of gelatin [8]. Sodium hexametaphosphate (SHP) is an amorphous polyphosphate sodium salt and a strong chelating agent, and the uniform charge distribution of SHP allows it to interact with positively charged proteins [9,10]. Therefore, SHP had been widely adopted to improve protein function. De et al. found that SHP had the ability to crosslink casein through calcium–casein phosphate complexes, and its calcium-binding capacity can influence the casein micelle stability in dairy products [10]. Shinde et al. found that the addition of sodium tripolyphosphate (STP) and SHP significantly improved the solubility, viscosity, foaming capacity, thermal stability, and gelling properties of milk protein concentrate [11]. Therefore, SHP was adopted to modify gelatin to improve the gelation behavior of gelatin in this study. Moreover, the function of transglutaminase (TG) is to catalyze the acyl transfer reaction between theε-amino group of lysine residues and the γ-amide group of glutamine residues. This process facilitates the formation of covalent bonds, which could further improve gelation effects and enhance gel strength by catalyzing protein polymerization [12]. Previous studies had shown that TG promoted intramolecular and intermolecular crosslinking reactions of gelatin molecules, which further altered the properties of gelatin [13]. Huang et al. found that TG could induce fish gelatin to form a network structure of denser gel and improve the properties of fish gelatin gel [14]. Enzymatic and chemical modification methods play a crucial role in protein engineering and functionalization [15]. SHP could interact with gelatin through ionic bond and hydrogen bonds interactions to stabilize the gelatin structure. Moreover, TG could catalyze the crosslinking of gelatin molecules to facilitate the formation of a stronger gel matrix. The presence of SHP may increase the availability of reactive sites for TG and enhance its catalytic activity. Therefore, we hypothesized that both SHP and TG could contribute to the viscosity and elasticity of the gelatin network during the gelation process. However, research on gelatin properties modified by both phosphates and TG remains scarcely reported. Therefore, the effects of different SHP concentrations (0, 0.4, 0.8, 1.2, 1.6, 2.0, 2.4, 2.8 mmol/L) on the texture, rheological behavior, water distribution, and microstructure of TG-crosslinked gelatin gels were investigated in this study.

## 2. Materials and Methods

### 2.1. Materials

SHP was ordered from Tianjin Deren Chemical Reagent Co., Ltd. (Tianjin, China). TG of 130 U/g was supplied by Jiangsu Yiming Biological Co., Ltd. (Taixing, China). Gelatin was obtained from Shangshui Fuyuan Gelatin Co., Ltd. (Zhoukou, China). Potassium bromide and acetic acid were ordered from Shanghai Yuan Ye Bio-Technology Co., Ltd. (Shanghai, China).

### 2.2. Composite Gelatin Gel Preparation

According to the method of Cen et al. [16] with slight modifications, the gelatin (3.5 g) was mixed with 96.5 mL of distilled water and heated at 45 °C for 20 min to prepare a gelatin solution of 3.5% (*w*/*v*). Different volumes of SHP were added to prepare gelatin–SHP solutions with the concentrations of 0, 0.8, 1.2, 1.6, 2.0, 2.4, and 3.2 mmol/L. The mixed solution pH was set at 4.8 using an acetic acid solution of 10%, and it was kept at 4 °C for 16–18 h for complete hydration. The mixture after TG (9 U/g gelatin) was added was heated at 45 °C for 2 h before keeping at 4 °C.

### 2.3. Determination of Water Distribution

The water distribution determination of the hydrogel was based on the method of Cheng et al. with some modifications [17]. An NMR analyzer (Niumag Analytical Instrument Co., Suzhou, China) was used to determine the water distribution of all the hydrogels. After keeping the different hydrogels at 4 °C for 16 h, 2 g of composite gelatin was put into a specialized glass tube and positioned at the center of the radio frequency coil. The different hydrogels were measured using the CPMG pulse sequence with the following parameters: sampling interval TW = 4000.0 ms, number of scans NS = 8, preamplification factor PRG = 1, echo time = 1.0 ms, and number of echoes = 10,000. The CPMG exponential decay curves were processed using Niumag NMR inversion software 2.0 to perform inversion fitting.

### 2.4. Texture Profile Analysis (TPA)

The measurement of the texture of the hydrogel was conducted according to the method of Yan et al. with minor modifications [18]. A TA-XT Express texture analyzer (Stable Micro Systems Ltd., Godalming, UK) was used to analyze the texture properties of TG-crosslinked gelatin gels with different concentrations of SHP. According to the method in Section 2.2, the above gel solution was put into cylindrical containers with 15 mm height and 18 mm diameter and stored at 4 °C for 12 h before testing. A test probe of P/36R and a trigger force of 5 g were selected, with a test speed of 1.00 mm/s and a posttest speed of 1.00 mm/s.

### 2.5. Rheological Properties Analysis

#### 2.5.1. Apparent Viscosity

The rheological properties of the hydrogel were determined based on the method reported by Tian et al. with some modifications [19]. A rheometer (Waters Corporation, Milford Massachusetts, USA) was used to analyze the apparent viscosity of the different hydrogels. The measurement gap was 1 mm with a parallel plate diameter of 40 mm at 20 °C and a shear rate ranging from 1 to 100 s^−1^.

#### 2.5.2. Frequency Sweep

A frequency sweep test of the different hydrogels was conducted using a rheometer (Waters Corporation, Milford Massachusetts, USA). The storage modulus (G′) and loss modulus (G″) were measured at a strain of 2%, with the frequency sweep ranging from 1 to 100 rad/s.

### 2.6. Intrinsic Fluorescence Spectra

The intrinsic fluorescence spectra were measured according to the method reported by Chen et al. [20]. A fluorescence spectrophotometer (Cary eclipse, Agilent, CA, USA) was adopted to measure the intrinsic fluorescence spectra of the different gels. The voltage was set at 700 V. The excitation wavelength was fixed at 280 nm, with the emission wavelength ranging from 300 nm to 470 nm.

### 2.7. Fourier Transform Infrared Spectroscopy (FTIR)

The FTIR of the hydrogel was carried out according to the method reported by Cheng et al. with some modifications [17]. An FTIR spectrometer (Bruker Optik GmbH, Ettlingen, Germany) was used to perform spectral scanning of the different microwave vacuum freeze-dried gels. The dried gels were uniformly mixed with KBr powder. After the above mixture was pressed into pellets, it was scanned ranging from 400 cm^−1^ to 4000 cm^−1^. The measurement was performed under a resolution of 4 cm^−1^ and 64 scans.

### 2.8. Scanning Electron Microscopy (SEM)

The microstructure of the hydrogel was measured based on Yan’s method [18]. A TM3030-Plus SEM (Hitachi High-Technologies Co., Tokyo, Japan) was used to observe the microstructure of the different gels. The holder with carbon conductive tape was used to fix the microwave vacuum freeze-dried gelatin, and the above gelatin surface was coated with gold. All the dried gels were scanned with 10 kV acceleration voltage at magnifications of 100× and 200× to observe their surface structure.

### 2.9. Statistical Analysis

All the experiments were conducted in triplicate, and the results were displayed as mean ± SD. The comparisons among different groups were conducted using one-way analysis of variance (*p* < 0.05), and the LSD test was selected to implement multiple comparisons. All the data were analyzed using SPSS 22.0, and all the figures were plotted by Origin 2020 software.

## 3. Results and Discussion

### 3.1. Analysis of Water Distribution

**Figure 1:**
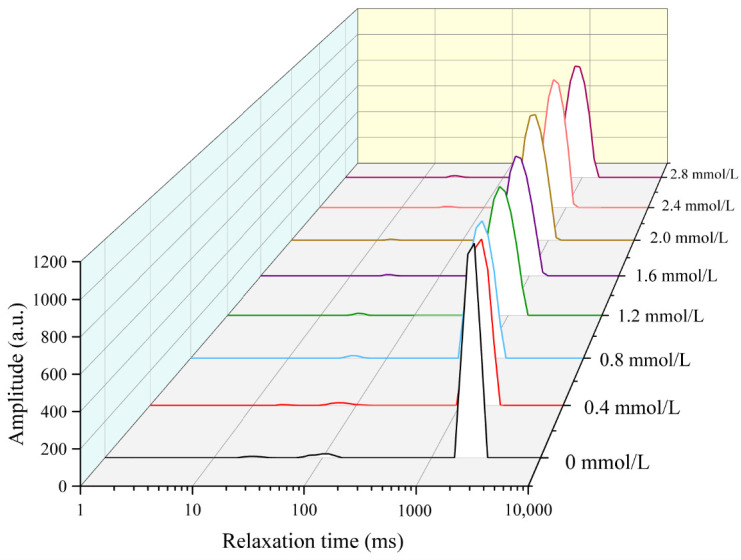
The water distribution of different TG-crosslinked gelatin hydrogels with different concentrations of SHP.

The water distribution of TG-crosslinked gelatin gels with different SHP addition levels is displayed in Figure 1. The water in all the different TG-crosslinked gelatin gels primarily consisted of free water. The relaxation time (T_2_) includes bound water (T_21_), free water (T_23_), and immobilized water (T_22_). The T_23,_ T_22_, and T_21_ of phosphorylated TG-crosslinked gelatin gels were remarkably lower than that of TG-crosslinked gelatin gels alone. A smaller T_2_ value indicates a more compact gel network structure with a lower water migration rate, which is consistent with the microscopic structure and texture of gels. The compact gel network structure could enhance the gel ability of capturing and adsorbing water [21]. When the SHP concentration was below 2.0 mmol/L, the T_2_ relaxation time of the TG-crosslinked gelatin gels showed a decreasing trend as the SHP addition level increased, indicating that the content of free water content was reduced by the modification of SHP. This was attributed to that the phosphate group introduction enhanced ionic interactions between PO_4_^3−^ and the NH_3_^+^ groups in the amino acids of gelatin molecules, leading to gelatin aggregation and reduced water mobility within the gel [16]. The binding of phosphates to gelatin molecules increased the charge density of gelatin molecules and enhanced the interactions between water and proteins, which increased the content of immobilized water in the TG-crosslinked gel [22]. It was also reported that sodium diphosphate and trisodium phosphate made a whole egg gel trap more immobilized water [23]. When the SHP concentration reached 2.0 mmol/L, the T_2_ relaxation time of the composite gelatin gels began to increase as SHP level increased. This might be attributed to that excessive phosphates enhanced electrostatic repulsion between gelatin molecules, which further increased the free water content between gelatin molecules [24].

### 3.2. Texture Analysis

The texture properties of different TG-crosslinked gelatin gels with different concentrations of SHP are shown in Table 1. The springiness, adhesiveness, and resilience of different TG-crosslinked gelatin gels with different concentrations of SHP did not have significant discrepancies (*p* < 0.05). The TG-crosslinked gelatin gel with an SHP concentration of 1.6 mmol/L exhibited the highest hardness (304.258 ± 11.167 g) and chewiness (366.916 ± 5.681 g). This was consistent with the highest G′ value that occurred in the TG-crosslinked gelatin with the SHP concentration of 1.6 mmol/L. This indicated that a suitable amount of SHP could enhance the hardness and chewiness of TG-crosslinked gelatin gels, which might be attributed to that the phosphate group introduced into gelatin molecules enhanced the aggregation of TG-crosslinked gelatin through ionic interactions with NH_3_^+^ groups in amino acids during gelation. The increase in the hardness of TG-crosslinked gelatin gels might also result from the reduction of free water content, which was in accordance with the result from the water distribution. The improvement in chewiness might be due to the increased crosslinking ratio between gelatin and SHP, forming a stronger gel network structure. The modification of fish gelatin with sodium tripolyphosphate also showed a similar tendency [16]. Additionally, appropriate phosphorylation pretreatment might have exposed more active sites to promote TG-induced gelatin crosslinking and the formation of a dense gel network. Zou et al. also found that phosphorylation pretreatment caused a more uniform and denser 3D network structure of TG-induced wheat gluten gels [25]. Furthermore, there were no significant differences in the hardness and chewiness of the TG-crosslinked gelatin gel with SHP concentrations of 1.2, 1.6, and 2.0 mmol/L (*p* > 0.05). However, excessive SHP negatively affected the chewiness and hardness of the TG-crosslinked gelatin, possibly due to the increased electrostatic repulsion between gelatin protein caused by excessive phosphates in gelatin. This led to the formation of rougher and less uniform gel networks, which further influenced the gel texture [24].

### 3.3. Apparent Viscosity Analysis

**Figure 2:**
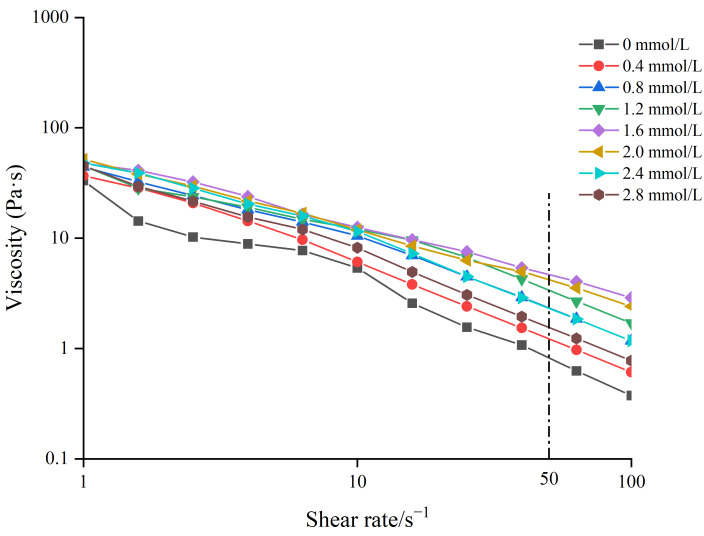
The apparent viscosity of TG-crosslinked gelatin with different SHP concentrations.

The apparent viscosities of different gelatin gels are shown in Figure 2. The apparent viscosity of different TG-crosslinked gelatin gels decreased when the shear rate increased, indicating that all the TG-crosslinked gelatin gels were non-Newtonian pseudoplastic fluid. Phosphorylation treatment did not alter the fluid properties of gelatin, as all the gelatin gels retained pseudoplastic behavior [25]. The viscosity at a shear rate of 50 s^−1^ denoted as η_50_ was closely correlated with the perceived thickness and viscosity of food [26]. The higher η_50_ value means a stronger shear resistance, which could maintain the structural integrity of food. Studies have shown that the addition of exogenous polysaccharides into low-fat cheese increased the η value and improved the texture and flavor stability of cheese [27].

The TG-crosslinked gelatin at the SHP concentration of 1.6 mmol/L exhibited the highest η_50_ value (4.81 Pa·s). The η_50_ of TG-crosslinked gelatin kept increasing before the SHP concentration reached 1.6 mmol/L, which might be due to the introduction of phosphates causing structure changes of TG-crosslinked gelatin molecules. The surface charge of TG-crosslinked gelatin was also altered by SHP, which further influenced the interaction between water and TG-crosslinked gelatin. This was consistent with the findings of Cen et al. who found that phosphorylation treatment increased the apparent viscosity of fish gelatin. The viscosity of the phosphorylated fish gelatin increased to 0.53 Pa·s, compared with the η_50_ of the control gel (0.156 Pa·s) [16]. Additionally, SHP modification could promote TG crosslinking and increase the molecular weight of gelatin, which increased the apparent viscosity of TG-crosslinked gelatin [25]. The decrease in the η_50_ of TG-crosslinked gelatin with the SHP concentration above 1.6 mmol/L might be attributed to excessive phosphates increasing the electrostatic repulsion of TG-crosslinked gelatin and promoting protein aggregation. The excessive phosphates induced reduced immobilized water content and increased free water content of TG-crosslinked gelatin and led to a reduced viscosity [28]. The result was in agreement with the result of water distribution in TG-crosslinked gelatin.

### 3.4. Frequency Sweep Analysis

**Figure 3:**
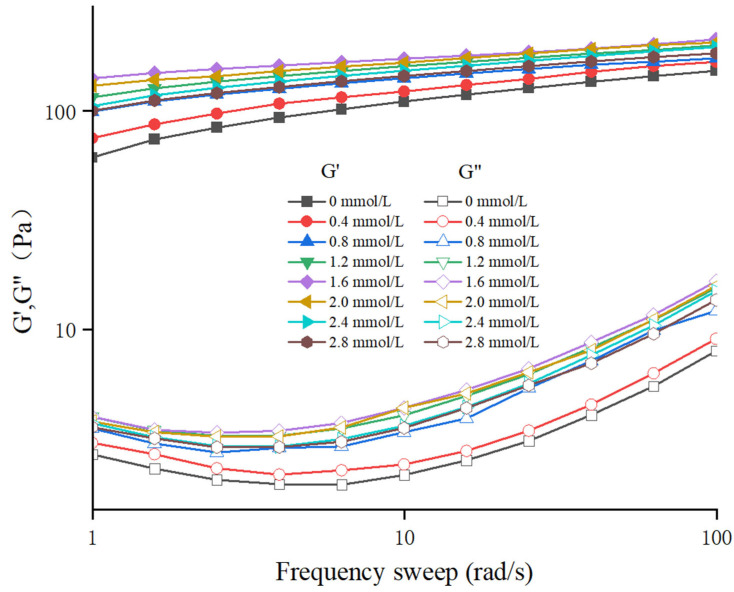
The frequence sweep of TG-crosslinked gelatin gels with different SHP concentrations.

**Figure 4:**
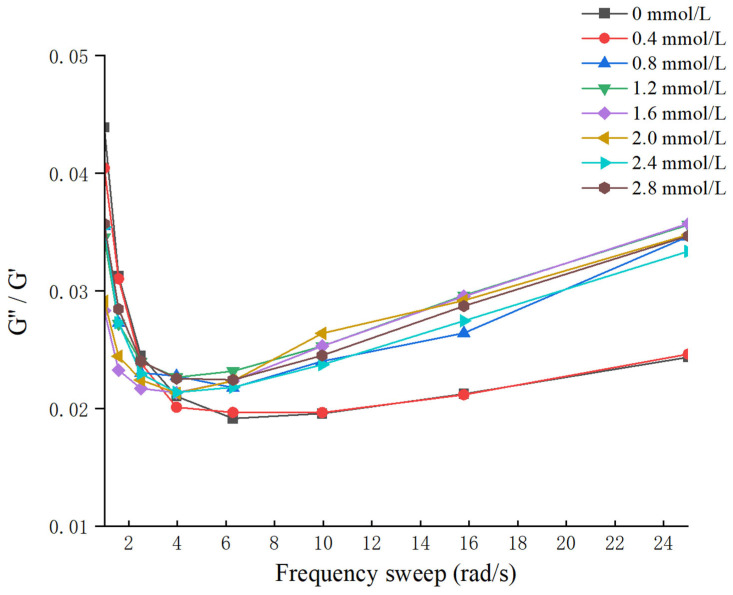
The G″/G′ values of TG-crosslinked gelatin gels with different SHP concentrations.

The frequency sweep result and G″/G′ values of different TG-crosslinked gelatin are shown in Figure 3 and Figure 4. The G′ values of all the TG-crosslinked gelatin gels in the whole frequency range were significantly higher than the G″ values, indicating that all the TG-crosslinked gelatin gels exhibited solid-like gel structure. A high G′ value indicates that the hydrogel has higher structural stability, which could resist more deformation and maintain food state [29]. The G′ and G″ of TG-crosslinked gelatin with SHP addition were both higher than that of non-phosphorylated TG-crosslinked gelatin, and the TG-crosslinked gelatin at the SHP concentration of 1.6 mmol/L exhibited the maximum values of G′ and G″, which contributed to the higher hardness and chewiness of TG-crosslinked gelatin. The G′ and G″ of TG-crosslinked gelatin increased before the SHP concentration reached 1.6 mmol/L, which was attributed to phosphate groups exposing hydrophobic amino acid residues and free sulfhydryl groups buried within gelatin molecules. This facilitated the formation of TG-crosslinked gelatin aggregates through non-covalent interactions (hydrophobic interaction and hydrogen bond) and intermolecular disulfide bonds, leading to the rearrangement of gelatin structure and the formation of a denser network structure [25]. This suggested that appropriate phosphorylation treatment reduced the water mobility within the TG-crosslinked gelatin network, which could make TG-crosslinked gelatin form a more uniform 3D network structure and enhance of the flowability and viscoelasticity of the gel system [25]. The G′ and G″ of TG-crosslinked gelatin with higher levels of SHP (>1.6 mmol/L) decreased due to the electrostatic repulsion between composite gelatin gel. However, the low G″/G′ values of all the TG-crosslinked gelatin gels at higher frequencies indicated that the gelatin gel network was weakened at higher frequencies [30].

### 3.5. Intrinsic Fluorescence Spectroscopy Analysis

**Figure 5:**
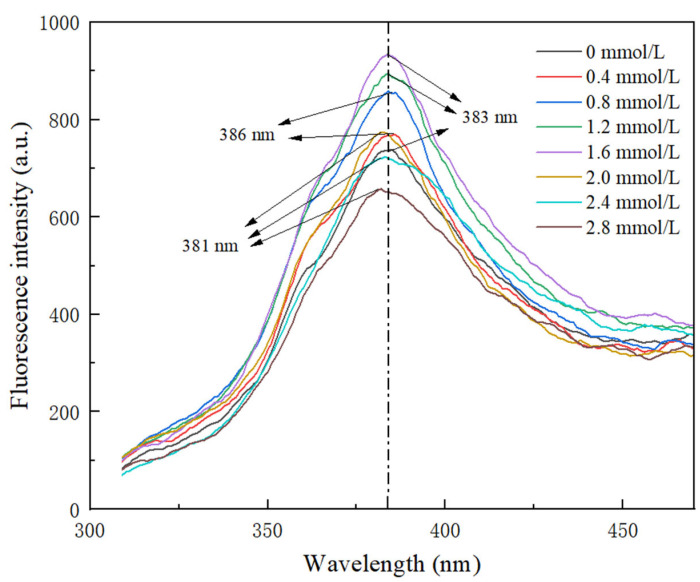
Intrinsic fluorescence spectrum of TG-crosslinked gelatin with different SHP concentrations.

The intrinsic fluorescence spectroscopy analysis of TG-crosslinked gelatin is shown in Figure 5. Tyrosine and phenylalanine in gelatin exhibit strong fluorescence intensity, which can reflect the changes in the gelatin tertiary structure [31]. As shown in Figure 5, with the increase in SHP concentration, the fluorescence intensity first increased and then decreased. The fluorescence intensity of TG-crosslinked gelatin at low SHP concentrations was significantly higher than that of TG-crosslinked gelatin without SHP. This might be because phosphorylation exposed more aromatic groups on the surface of the gelatin. Moreover, phosphorylation pretreatment exposed more active sites in gelatin susceptible to TG, which increased the degree of protein crosslinking and exposed more hydrophobic amino acids. After the SHP concentration reached 1.6 mmol/L, the fluorescence intensity of TG-crosslinked gelatin decreased with the increasing SHP concentration. Compared with the λ_max_ (383 nm) of TG-crosslinked gelatin without SHP, the λ_max_ of gelatin with a higher concentration of SHP (>1.6 mmol/L) showed blue shift. Compared with the TG-crosslinked gelatin alone, the fluorescence intensity of TG-crosslinked gelatin with 0.4–2.4 mmol/L SHP increased from 720–769. This might be because the phosphate groups enhanced electrostatic repulsion between TG-crosslinked gelatin, which caused the unfolding of TG-crosslinked gelatin chains and the exposing of more hydrophobic regions. These hydrophobic groups formed aggregates via hydrophobic interactions and became buried within TG-crosslinked gelatin, resulting in the decreased fluorescence intensity of TG-crosslinked gelatin at a high SHP concentration [25]. Zhang et al. also reported that the phosphorylation degree of soy protein isolate decreased at higher concentrations of sodium tripolyphosphate [24].

### 3.6. FTIR

**Figure 6:**
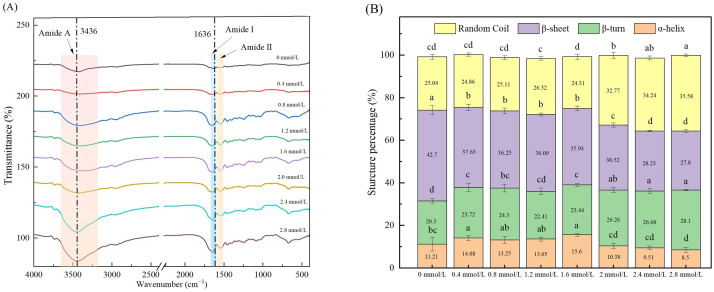
FTIR spectroscopy (**A**) and secondary structure content (**B**) of TG-crosslinked gelatin with different SHP concentrations. Different letters indicated significant differences.

The FTIR spectra of TG-crosslinked gelatin with different SHP concentrations are shown in Figure 6A. The second structure of gelatin with different SHP concentrations is displayed in Figure 6B. The Amide A (3600–3100 cm^−1^) of protein is related to the stretching vibrations of intramolecular N-H bonds [32]. The Amide A band corresponded to the coupling of hydrogen bonding and NH-stretching vibrations. The absorption peak of Amide A of TG-crosslinked gelatin without SHP modification appeared at 3436 cm^−1^. The Amide A absorption peaks of TG-crosslinked gelatin with SHP concentrations of 0.4, 0.6, 0.8, 1.2, 1.6, 2.0, and 2.4 mmol/L were 3418 cm^−1^, 3421 cm^−1^, 3420 cm^−1^, 3340 cm^−1^, 3430 cm^−1^, 3445 cm^−1^, and 3442 cm^−1^, respectively.

Before the SHP concentration reached 1.6 mmol/L, the Amide A of TG-crosslinked gelatin displayed a red shift, which was attributed to the formation of more hydrogen bonds between -COOH/-OH groups and -NH_2_ residues in gelatin [32]. After the SHP concentration exceeded 1.6 mmol/L, the Amide A of TG-crosslinked gelatin indicated a blue shift, which was due to the electrostatic interactions between positively charged NH_4_^+^ groups in gelatin and negatively charged PO_4_^3−^ groups in SHP. Sow et al. reported that electrostatic interactions affected molecular structures by shortening the bond length of N-H groups and led to the increased wavenumber of N-H groups [33]. The stretching vibrations of C=O and C=N bonds was associated with the Amide I band (1700–1600 cm^−1^) [34]. The Amide I band (1700–1600 cm^−1^) is always used for analyzing the secondary structure of proteins [35], which was related with α-helices, random coils, β-sheets, and β-turns [36]. The Amide I absorption peak of TG-crosslinked gelatin without SHP modification was at 1636 cm^−1^. The Amide I absorption intensity of TG-crosslinked gelatin with SHP was higher than that of TG-crosslinked gelatin alone. The Amide I absorption peaks of TG-crosslinked gelatin with the SHP concentrations of 0.4, 0.6, 0.8, 1.2, 1.6, 2.0, and 2.4 mmol/L were 1643 cm^−1^, 1649 cm^−1^, 1649 cm^−1^, 1652 cm^−1^, 1643 cm^−1^, 1643 cm^−1^, and 1643 cm^−1^, respectively. Compared to TG-crosslinked gelatin without SHP modification, all the TG-crosslinked gelatin gels with SHP addition exhibited a blue shift of the Amide I band, which might be attributed to the electrostatic interactions caused by the introduced phosphate groups. The Amide II band (1500–1550 cm^−1^) represents the vibration of N-H deformation. Compared with the absorption peak (1541 cm^−1^) of TG-crosslinked gelatin alone, the TG-crosslinked gelatin with all the different levels of SHP showed a blue shift. As can be seen in Figure 6B, when the SHP concentration exceeds 1.6 mmol/L, the content of random coiling of TG-crosslinked gelatin gradually increased, suggesting that the ordered structure of the secondary structure of TG-crosslinked gelatin was disrupted by the modification of SHP. Moreover, the β-turn content of all the TG-crosslinked gelatin gels with SHP modification was lower than that of TG-crosslinked gelatin without SHP, which was consistent with the rough and wrinkled structure of TG-crosslinked gelatin with high levels of SHP observed in SEM. Suitable levels of phosphorylation might strengthen the random coil structure of gelatin through increased repulsions between charged residues of gelatin and further improve the gelatin gel properties.

### 3.7. SEM Analysis

**Figure 7:**
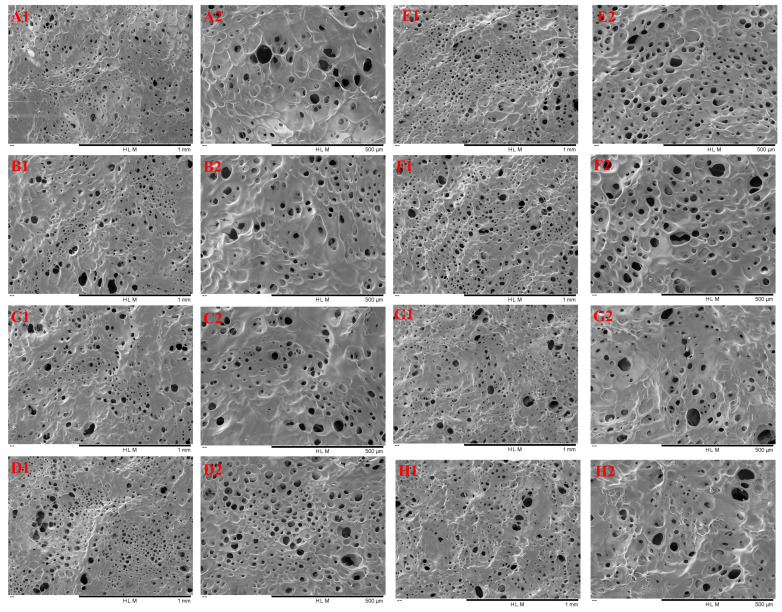
SEM image of TG-crosslinked gelatin with different SHP concentrations. Note: (**A**)–(**H**) represent the TG-crosslinked gelatin with SHP concentrations of 0, 0.4, 0.8, 1.2, 1.6, 2.0, 2.4, and 2.8 mmol/L, respectively. Subscripts 1 and 2 indicate magnifications of 100× and 200×, respectively.

The microstructures of TG-crosslinked gelatin with different SHP concentrations are shown in Figure 7. The pore size of TG-crosslinked gelatin with different SHP concentrations is displayed in Table 2. All the TG-crosslinked gelatin gels exhibited the structure of honeycomb, which displayed the typical gelatin network structure. Compared to the TG-crosslinked gelatin with SHP modification, the TG-crosslinked gelatin without SHP modification showed a looser and uneven network with larger voids. In contrast, the phosphorylated gelatin gels had a compact and ordered network, which might be attributed to the involvement of phosphate groups in network formation. The TG-crosslinked gelatin with low SHP concentrations exhibited a denser, smoother, and more uniform structure. As the SHP in lower concentrations promoted the order folding and unfolding of GE and TG-induced crosslinking, more hydrophobic groups and free sulfhydryl groups were exposed on the surface of GE. This facilitated disulfide bond and hydrophobic interactions and enabled the crosslinking reactions of TG to form a more uniform and denser gel network structure [25], which was related with the decrease in T_2_ and free water content in TG-crosslinked gelatin with SHP. The hydrogel network at the SHP concentration of 1.6 mmol/L exhibited a porous, smooth, and dense structure. The pore size of TG-crosslinked gelatin with SHP showed a decreasing tendency before the SHP reached 1.6 mmol/L, which was in agreement with the change of T_2_. This might be attributed to suitable levels of SHP making the gel network become denser. However, when the SHP concentration exceeded 2.0 mmol/L, the network of TG-crosslinked gelatin became wrinkled, and its surface roughness significantly increased. This could be due to excessive phosphate groups increasing the electrostatic repulsion between TG-crosslinked gelatin and causing gelatin chains to aggregate into bundles, which disrupted the gelatin structure and resulted in a rougher and more irregular network. These findings were consistent with the textural properties of TG-crosslinked gelatin after phosphorylation pretreatment. Therefore, suitable SHP concentration could significantly improve the microstructure network of TG-induced gelatin gels.

## 4. Conclusions

This study combined TG and different SHP concentration to modify gelatin gels. When the SHP concentration was below 2.0 mmol/L, the water mobility in TG-crosslinked gelatin decreased. A suitable level of SHP could enhance the hardness and chewiness of TG-crosslinked gelatin gels. SHP modification altered the tertiary structure of TG-crosslinked gelatin. All the TG-crosslinked gelatin gels with SHP addition exhibited solid-like gel structures. The hydrogel network at the SHP concentration of 1.6 mmol/L exhibited a porous, smooth, and dense structure. Future research should focus on the effects of modified gelatin gel on the encapsulation efficiency of bioactive substances and probiotics. Moreover, the digestion characteristics of the compounds and probiotics encapsulated by modified gelatin in vitro and in vivo should also be studied. This research establishes a good foundation for developing gelatin-based gel systems.

## Figures and Tables

**Table 1:** The texture properties of TG-crosslinked gelatin with different SHP concentrations.

SHMP/mmol·L^−1^	Hardness (g)	Springiness	Cohesiveness	Gelatinous	Chewiness	Resilience
0	215.382 ± 7.313 ^c^	0.960 ± 0.018 ^a^	0.913 ± 0.009 ^a^	194.154 ± 10.080 ^c^	308.788 ± 3.872 ^d^	0.775 ± 0.010 ^a^
0.4	252.981 ± 11.283 ^b^	0.963 ± 0.024 ^a^	0.907 ± 0.013 ^a^	218.869 ± 1.217 ^b^	347.248 ± 6.210 ^b^	0.780 ± 0.001 ^a^
0.8	260.655 ± 5.304 ^b^	0.964 ± 0.008 ^a^	0.905 ± 0.007 ^a^	220.547 ± 1.447 ^b^	349.897 ± 3.285 ^b^	0.785 ± 0.144 ^a^
1.2	302.969 ± 18.449 ^a^	0.970 ± 0.007 ^a^	0.909 ± 0.006 ^a^	236.068 ± 7.020 ^a^	362.377 ± 13.135 ^a^	0.789 ± 0.008 ^a^
1.6	304.258 ± 11.167 ^a^	0.965 ± 0.025 ^a^	0.909 ± 0.007 ^a^	240.287 ± 6.681 ^a^	366.916 ± 5.681 ^a^	0.783 ± 0.007 ^a^
2.0	299.601 ± 5.150 ^a^	0.970 ± 0.017 ^a^	0.919 ± 0.009 ^a^	234.701 ± 4.796 ^a^	362.991 ± 8.137 ^a^	0.772 ± 0.004 ^a^
2.4	247.144 ± 16.343 ^b^	0.965 ± 0.119 ^a^	0.915 ± 0.006 ^a^	215.880 ± 10.669 ^b^	330.297 ± 8.152 ^c^	0.782 ± 0.006 ^a^
2.8	205.945 ± 10.304 ^c^	0.968 ± 0.183 ^a^	0.916 ± 0.009 ^a^	193.291 ± 8.756 ^c^	300.391 ± 7.706 ^d^	0.776 ± 0.214 ^a^

Note: The above data were presented as mean ± SD; Different letters in the same column indicated significant differences (*p* < 0.05).

**Table 2:** The pore size of TG-crosslinked gelatin with different SHP concentrations.

SHP Concentrations (mmol/L)	0	0.4	0.8	1.2	1.6	2.0	2.4	2.8
Pore size (μm)	54.0 ± 1.87 ^a^	51.4 ± 1.94 ^b^	51.2 ± 1.3 ^b^	48.4 ± 1.14 ^d^	31.2 ± 1.09 ^e^	47.8 ± 1.64 ^d^	50.4 ± 1.67 ^bc^	48.8 ± 1.92 ^cd^

Different superscript letters indicated significant differences.

## Data Availability

The original contributions presented in the study are included in the article, further inquiries can be directed to the corresponding author.
